# Impairment in emotion perception from body movements in individuals with bipolar I and bipolar II disorder is associated with functional capacity

**DOI:** 10.1186/s40345-017-0083-7

**Published:** 2017-05-17

**Authors:** Anja Vaskinn, Trine Vik Lagerberg, Thomas D. Bjella, Carmen Simonsen, Ole A. Andreassen, Torill Ueland, Kjetil Sundet

**Affiliations:** 1 Department of Psychology, University of Oslo, P.O. Box 1094, Blindern, 0317 Oslo, Norway; 2 NORMENT KG Jebsen Centre for Psychosis Research, Oslo University Hospital, Oslo, Norway; 3 Early Intervention in Psychosis Advisory Unit, Oslo University Hospital, Oslo, Norway; 4 Department of Mental Health and Addiction, Institute of Clinical Medicine, University of Oslo, Oslo, Norway

**Keywords:** Bipolar disorder, Social cognition, Emotion perception, Body language, Cognition, Psychosis, Functioning, Functional capacity

## Abstract

**Background:**

Individuals with bipolar disorder present with moderate impairments in social cognition during the euthymic state. The impairment extends to theory of mind and to the perception of emotion in faces and voices, but it is unclear if emotion perception from body movements is affected. The main aim of this study was to examine if participants with bipolar disorder perform worse than healthy control participants on a task using point-light displays of human full figures moving in a manner indicative of a basic emotion (angry, happy, sad, fearful, neutral/no emotion). A secondary research question was whether diagnostic subtypes (bipolar I, bipolar II) and history of psychosis impacted on this type of emotion perception. Finally, symptomatic, neurocognitive, and functional correlates of emotion perception from body movements were investigated.

**Methods:**

Fifty-three individuals with bipolar I (*n* = 29) or bipolar II (*n* = 24) disorder, and 84 healthy control participants were assessed for emotion perception from body movements. The bipolar group also underwent clinical, cognitive, and functional assessment. Research questions were analyzed using analyses of variance and bivariate correlations.

**Results:**

The bipolar disorder group differed significantly from healthy control participants for emotion perception from body movements (Cohen’s *d* = 0.40). Analyses of variance yielded no effects of sex, diagnostic subtype (bipolar I, bipolar II), or history of psychosis. There was an effect of emotion, indicating that some emotions are easier to recognize. The lack of a significant group × emotion interaction effect points, however, to this being so regardless of the presence of bipolar disorder. Performance was unrelated to manic and depressive symptom load but showed significant associations with neurocognition and functional capacity.

**Conclusions:**

Individuals with bipolar disorder had a small but significant impairment in the ability to perceive emotions from body movement. The impairment was global, i.e., affecting all emotions and equally present for males and females. The impairment was associated with neurocognition and functional capacity, but not symptom load. Our findings identify pathopsychological factors underlying the functional impairment in bipolar disorder and suggest the consideration of social cognition training as part of the treatment for bipolar disorder.

## Background

Although traditionally conceptualized as an affective disorder, bipolar disorder (BD) is increasingly recognized as a mental illness also characterized by cognitive impairments. Whereas the diagnostic criteria continue to highlight changes in mood and behavior, research shows that individuals with BD have cognitive impairments (Bora et al. 2009; Cardenas et al. 2016) that impact cross-sectionally and longitudinally on function in everyday life (Baune and Malhi 2015). The cognitive impairments are less pronounced than in schizophrenia but present in the same domains (Bortolato et al. 2015). Prevalence rates of clinically relevant cognitive impairment in BD vary across studies and domains, but exceed the rates seen in the general population (Cullen et al. 2016).

Whereas neurocognition has been thoroughly studied, social cognition in BD has received less attention. Social cognition has been defined as “the mental operations that underlie social interactions, including perceiving, interpreting and generating responses to the intentions, dispositions and behaviors of others” (Green et al. 2008). Social cognition encompasses different domains (Pinkham 2014) of which emotion perception and theory of mind (ToM, i.e., the ability to infer the intentions, dispositions, or beliefs of others) have been the most commonly investigated in BD. Individuals with BD present with deficits for both domains, across illness phase, i.e., in manic, depressed, or euthymic states (Samamé 2013). Firstly, according to a systematic review, deficits are more pronounced for ToM (*d* = 0.5–0.8) than for emotion perception (*d* < 0.5), albeit significantly for both domains (Samamé et al. 2012), in the euthymic phase. Moreover, a meta-analysis (Bora et al. 2016) found ToM deficits to be significantly more pronounced (*d* = 1.23) in acute phases (manic or depressed mood). However, reviews and meta-analyses show that individuals with BD perform better than persons diagnosed with schizophrenia for both facial emotion perception and ToM (Bora and Pantelis 2016), especially for complex ToM tasks of a more ecological valid nature, where individuals with schizophrenia evidence large deficits (Mitchell and Young 2014).

Within the BD population, results are mixed when it comes to cognitive differences between diagnostic subtypes (BD I versus BD II) and the effect of history of psychosis on cognition (Lewandowski et al. 2011). For example, in our previous research, we found neurocognitive performance to depend on history of psychosis, not diagnostic subtype, in individuals with established BD I or II disorder (Simonsen et al. 2011), but detected no neurocognitive differences between psychotic and non-psychotic subgroups with first-treatment BD I (Demmo et al. 2016). For social cognition, there are also divergent findings. Some studies find worse performance in BD I compared to BD II (Lembke and Ketter 2002; Derntl et al. 2009); others find similar performance (Martino et al. 2011). A meta-analysis of ToM (Bora et al. 2016) found the same magnitude of impairment for BD I as for the whole BD spectrum, as well as a non-significant, trend-level association between history of psychosis and ToM performance. This unresolved issue is probably due to the large heterogeneity within the BD population, as well as in study samples.

A key factor related to daily social function is the perception of emotions in other people. We communicate emotions by the way we move and talk; and therefore also perceive emotions in different modalities. Visual emotion signals can be conveyed in facial expressions (facial emotion perception) or body movement (body language), whereas auditory signals are expressed through prosodic information in vocal expressions (auditory emotion perception). Most studies have used pictures of faces expressing one of the six basic emotions to assess emotion perception. Persons with BD have a moderate deficit in facial emotion perception (Kohler et al. 2011). Findings for auditory emotion perception have been mixed. Some studies have found intact auditory emotion perception for BD I (Vaskinn et al. 2007; Hoertnagel et al. 2015), whereas others have found limited impairments for some emotions for either females (Bozikas et al. 2007) or males (van Reenen and Rossell 2013) with the disorder. To the best of our knowledge, there are no studies of emotion perception from body movement in individuals with BD. This can be assessed with so-called point-light displays (PLD). In PLD tasks (Johansson 1973) a light is connected to different parts of the human body, or the face, while the person is filmed when moving in a dark room. Perceivers are able to infer sex (Kozlowski and Cutting 1977), personality traits (Gunns et al. 2001), and emotions (Heberlein et al. 2004) from PLDs. We have previously shown that individuals with schizophrenia, regardless of sex, have a global deficit in the perception of emotions from body movement (Vaskinn et al. 2016), in line with other studies (Couture et al. 2010; Kern et al. 2013; Okruszek et al. 2015).

Being able to detect the emotional state of others has adaptive interpersonal value. Because BD is associated with social functional impairments (Simon 2003; Sanchez-Moreno et al. 2009), it is of interest to investigate if individuals with the illness have difficulties detecting emotions from body movements, and if this is related to functioning. Only a few studies have investigated the relationship between social cognition and functioning in BD. One of these found larger social cognition deficits in a low-functioning compared to a higher functioning group (Lahera et al. 2012). In another study, social/emotional processing predicted functional capacity, i.e., the ability to perform functional tasks in the test laboratory (Thaler et al. 2014).

In the current study, we have three research aims. Our first research question relates to whether individuals with BD have impairments in emotion perception from body movement (or body language). We investigate case-control differences for overall body language reading, for body language reading of specific emotions, and if sex differences are present. We expect that the BD group will perform significantly worse than the HC group, but anticipate no sex differences based on our previous findings using the same test (Vaskinn et al. 2016). In our second research aim, we investigate if there are differences between participants with BD I and BD II, and if history of psychosis is associated with body language reading performance. Based on the mixed results in the cognitive literature, we make no hypotheses for this research aim. The third research aim is to investigate symptomatic, neurocognitive, and functional correlates of body language reading performance in participants with BD. Participants at our research center are only partly and only mildly symptomatic, and therefore no associations with symptom load are expected. Because cognition has been shown to be associated with functional outcome in bipolar disorder, we predict significant relations between body language reading and measures of functioning.

## Methods

### Design and participants

This cross-sectional study was conducted as part of the Thematically Organized Psychosis (TOP) study at the KG Jebsen NORMENT Centre for Psychosis Research at the University of Oslo, Norway, from 2013 to 2015. Individuals with DSM-IV BD I or BD II disorder as well as HC were included. All participants had an IQ ≥ 70 as assessed with the Wechsler Abbreviated Scale of Intelligence (WASI) (Wechsler 2007). The clinical group was recruited from inpatient and outpatient units at hospitals in the greater Oslo area and assessed in a clinically stable state. Diagnostic assessments were undertaken by trained clinicians using the SCID interview (First et al. 1995). HC were randomly selected from national statistical records and invited to participation by letter. Before inclusion, HC were screened for symptoms of severe mental illness using the Primary Care Evaluation of Mental Disorders interview (PRIME-MD: Spitzer et al. 1994) and excluded from the study if mental, neurological, or somatic disorder was confirmed or suspected. The study has been approved by the Regional Committee for Medical Research Ethics and the Norwegian Data Inspectorate. All participants signed informed consent after receiving oral and written information.

## Materials

### Clinical assessments

Global symptoms were assessed with the Global Assessment of Functioning-Split version (Pedersen et al. 2007), symptoms subscale. Depressive symptoms were measured with the Inventory of Depressive Symptomatology (IDS-C: Rush et al. 1996) and manic symptoms were assessed with the Young Mania Rating Scale (YMRS: Young et al. 1978). The mean depression score of 16.3 (SD = 10.1) indicates mild depression, whereas the mean mania score of 2.4 (SD = 3.9) indicates a normal (non-manic state). 43% (*n* = 23) of the sample was fully euthymic at the time of testing (IDS-C ≤ 12; YMRS < 8: Tohen et al. 2009). The defined daily dose (DDD) is the “assumed average maintenance dose per day for a psychopharmacological drug used for its main indication in adults” (World Health Organization 2016). The DDD for antipsychotic, antiepileptic, and antidepressant treatment, as well as for lithium treatment was calculated.

### Assessment of emotion perception

Body language reading ability was assessed using human full-figure PLDs. We used Heberlein et al. (2004) stimuli, as adapted by Couture et al. (2010), but with Norwegian norms (Vaskinn et al. 2016). This test has been referred to as Emotional Biological Motion, or EmoBio, and consists of 22 short clips of PLD walkers. The clips were shown on a computer screen, and participants indicated on a piece of paper which emotion was depicted by ticking the preferred alternative. Emotions were angry, happy, sad, fearful, or neutral/no emotion. The standard proportional scoring method was utilized: each response is given credit based on the proportion of healthy control participants giving that response. If 60% of healthy control participants say “happy,” 25% say “sad,” and 15% say “neutral,” a “happy” response is scored 1 (60/60), a “sad” response is scored 0.42 (25/60), and a “neutral” response is scored 0.25 (15/60). This scoring method accepts a certain degree of variability as normal. Both the overall total EmoBio score as well as the EmoBio scores for each of the five emotion categories are used in the current study.

### Assessment of cognition

All participants underwent assessments with a battery consisting of neuropsychological and social cognitive tests. Current IQ was measured with the 2-test WASI (Wechsler 2007). Neurocognition was measured with the MATRICS Consensus Cognitive Battery (MCCB; Nuechterlein et al. 2009; Mohn et al. 2012). The MCCB includes the following neuropsychological tests (domain assessed in parentheses): Trail Making Test A, BACS Symbol Coding and Category Fluency (speed of processing), Spatial Span and Letter Number Span (working memory), NAB Mazes (reasoning and problem-solving), CPT-IP (attention), Hopkins Verbal Learning Test and Brief Visual Memory Test (learning and memory). The MCCB social cognition subtest was excluded because we wanted to use the MCCB as a measure of non-social cognition. The test battery was administered by clinical psychologists trained by specialists in clinical neuropsychology.

### Functional assessment

Global functioning was assessed with the Global Assessment of Functioning-Split version (Pedersen et al. 2007), function subscale. Self-reported social functioning was measured with the Social Functioning Scale (Birchwood et al. 1990) which was developed for schizophrenia, but has been shown to be suitable for use in BD research (Hellvin et al. 2010). Functional capacity was assessed with the University of California San Diego Performance-Based Skills Assessment, brief version (UPSA-B; Mausbach et al. 2007). This is a role-play test where the participant is presented with props and asked to solve everyday tasks. Two modules are administered: Financial Skills where tasks include counting change and paying bills, and Communication Skills where a telephone is used to call for help in an emergency and to change a doctor’s appointment. The UPSA-B has been adapted to Norwegian conditions and approved by the original developers. In the Communication Skills domain, a check is used in the original version. As checks are no longer used in Norway, this was exchanged with an invoice. Further, Norwegian currency and telephone numbers were used.

### Statistical analysis

Analyses were done using The Statistical Package for the Social Sciences (IBM SPSS Statistics for Windows, Version 22.0, IBM Corp, Armonk, NY). Normality of distributed scores was investigated through histograms and skewness indices. All EmoBio measures had negative skewness values with scores clustering at the high end. The Kolmogorov–Smirnov statistic was significant (*p* < 0.001) for all measures. The EmoBio data were therefore reflected and transformed (log10). An initial univariate analysis of variance ANOVA looked at overall case-control differences for the total EmoBio score. Thereafter, a 2 × 2 × 5 repeated measures ANOVA (or mixed between within-subjects ANOVA) of the effect of diagnostic group (HC/BD) and sex (male/female) on body language reading was conducted. The 5 EmoBio scores (angry, happy, sad, fearful, neutral) were entered as dependent variables (within-subjects factor). Diagnostic group (HC/BD) and sex (male or female) were the between-subject factors. The impact of history of psychosis and diagnostic subtype (BD I or BD II) on body language reading within the BD sample was examined with a univariate ANOVA with EmoBio total (log transformed) entered as the dependent variable, and history of psychosis and diagnostic subtype as fixed factors. Associations between body language reading and symptoms (YMRS, IDS-C, and GAF-s), neurocognition (MCCB), and functioning (GAF-f, SFS, UPSA-B) were investigated using bivariate correlations (Pearson’s *r*). The effect of psychopharmacological treatment on emotion perception from body movements was examined by conducting bivariate correlations (Pearson’s *r*) between EmoBio total (log-transformed) and DDD of the compound in question, in participants who used it.

## Results

Fifty-three individuals with DSM-IV BD I (*n* = 29) or BD II (*n* = 24) disorder were included, along with 84 HC whose EmoBio data were also used in a previous paper (Vaskinn et al. 2016). Within the BD sample, 27 participants had a history of psychosis (BD I *n* = 24; BD II *n* = 3), whereas 26 (BD I *n* = 5; BD II *n* = 21) had never experienced psychotic symptoms. See Table 1 for demographic and clinical information. Results on the EmoBio test are shown in Table 2.

**Table 1:** Demographics in participants with bipolar disorder (BD) and healthy participants (HC), and clinical features in participants with bipolar disorder (BD)

	BD	HC	Statistic	
	(*n* = 53)	(*n* = 84)		
	Mean (SD)	Mean (SD)	Value	Sig
Age	34.3 (13.3)	30.8 (8.1)	*t* = 1.96	ns
Gender (males/females)	17/36	51/33	*x* ^2^ = 10.66	p = 0.001
WASI IQ	107.6 (10.5)	112.0 (11.5)	*t* = 2.27	p = 0.025
GAF-symptoms	61.1 (9.7)	–	–	–
GAF-function	58.3 (11.9)	–	–	–
YMRS	2.4 (3.9)	–	–	–
IDS-C	16.3 (10.1)^a^	–	–	–
Medication^b^				
Antipsychotic	0.94 (0.70)	–	–	–
	*n* = 28 (53%)			
Antiepileptic	1.07 (0.27)	–	–	–
	*n* = 12 (23%)			
Lithium	0.98 (0.48)	–	–	–
	*n* = 16 (30%)			
Antidepressant	2.11 (2.11)	–	–	–
	*n* = 11 (21%)			

*WASI* Wechsler Abbreviated Scale of Intelligence, *GAF* Global Assessment of Functioning, *YMRS* Young Mania Rating Scale, *IDS*-*C* Inventory of Depressive Symptoms-Clinician rated

^a^ *n* = 48 due to missing data

^b^Average defined daily dose

**Table 2:** Scores on the body language reading test (point-light walkers) in participants with bipolar disorder (BD) and healthy participants (HC) across sex

	BD Mean (SD)			HC Mean (SD)			Statistics
	Total *n* = 53	Males *n* = 17	Females *n* = 36	Total *n* = 84	Males *n* = 51	Females *n* = 33	
EmoBio total	0.83 (0.12)	0.81 (0.13)	0.84 (0.12)	0.87 (0.08)	0.86 (0.08)	0.88 (0.09)	ANOVA: Group: *F* = 4.85, *p* = 0.029
EmoBio angry	0.72 (0.24)	0.71 (0.24)	0.72 (0.24)	0.81 (0.17)	0.81 (0.15)	0.81 (0.21)	ANOVA: Group: *F* = 5.06, *p* = 0.026 Sex: *F* = 1.77, ns Emotion: *F* = 14.92, *p* < 0.001 All interaction effects: ns
EmoBio happy	0.89 (0.13)	0.85 (0.15)	0.91 (0.12)	0.91 (0.11)	0.92 (0.09)	0.89 (0.13)	
EmoBio sad	0.84 (0.20)	0.84 (0.22)	0.84 (0.19)	0.87 (0.17)	0.85 (0.19)	0.90 (0.12)	
EmoBio fearful	0.80 (0.25)	0.84 (0.25)	0.79 (0.25)	0.82 (0.18)	0.82 (0.16)	0.82 (0.21)	
EmoBio neutral	0.89 (0.18)	0.82 (0.20)	0.92 (0.16)	0.92 (0.13)	0.90 (0.12)	0.96 (0.12)	

EmoBio: Emotional Biological Motion

There was a small, but significant difference between HC and BD participants for the total EmoBio score (*F*_(1,136)_ = 4.85, *p* = 0.029, *η* ^2^ = 0.03). The Levene test statistic (4.61, *p* = 0.034) indicated unequal variances across groups, but the robust Welch test for equality of means still yielded a statistically significant group difference (4.16, *p* = 0.045). The effect size (Cohen’s *d*) using the pooled standard deviation was 0.40. Significant group differences were seen in the sex distribution and for intellectual abilities. The diagnostic group difference in EmoBio performance remained significant after controlling for sex as a covariate, and sex was therefore kept as a separate independent variable in the subsequent analysis. Subtle reductions in IQ are an attribute of BD (Cardenas et al. 2016) and do not appear by chance due to our study design. We therefore refrained from controlling for IQ (Dennis et al. 2009).

**Fig. 1:**
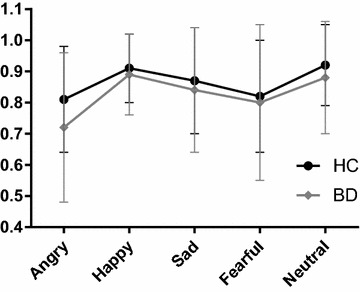
Scores on the body language reading test of sad, fearful, happy, angry, and neutral point-light walkers in participants with bipolar disorder (BD) and healthy participants (HC). Result summary: Individuals with bipolar disorder had a small, but statistically significant impairment in the ability to perceive emotions from body movement. The impairment was global and affected all emotions to the same extent. *Error bars* correspond to standard deviations for the two groups

In the repeated measures ANOVA across the five emotions, the main between-subject effect of diagnostic group was significant (*F*_(1,133)_ = 5.06, *p* = 0.026, *η* ^2^ = 0.04), with participants with BD performing significantly worse than HC, corroborating the results of the first ANOVA for the total score. Further, the main within-subject effect of emotion was significant (*F*_(4,130)_ = 12.38, Wilk’s Lambda = 0.72, *p* < 0.001, *η* ^2^ = 0.28), indicating that performance differed across emotion. This is shown in Fig. 1 where the performance of the two groups (HC and BD) is depicted. The group × emotion interaction effect was non-significant (*F*_(4,130)_ = 1.23, Wilk’s Lambda = 0.96, *p* = 0.300, *η* ^2^ = 0.04), reflected in the similar profile shape of body language reading performance for HC and BD participants in Fig. 1. In other words, individuals with BD are not disproportionally impaired on any one emotion compared to HC. Neither the main between-subject effect of sex (*F*_(1,133)_ = 1.77, *p* = 0.186, *η* ^2^ = 0.01) nor any of the other interaction effects were significant.

The univariate ANOVA within the BD sample yielded no significant effect of history of psychosis (*F*_(1,49)_ = 0.30, *p* = 0.588, *η* ^2^ = 0.01) or diagnostic subtype (*F*_(1,49)_ = 0.05, *p* = 0.826, *η* ^2^ < 0.001) on body language reading performance.

Bivariate associations between body language reading and measures of symptoms, neurocognition and functioning in participants with BD were mostly small and non-significant (see Table 3). There were two exceptions. EmoBio was moderately associated with neurocognition (MCCB, *r* = 0.33), and strongly associated with functional capacity (UPSA-BN, *r* = 0.48).

**Table 3:** Bivariate associations (Pearson’s *r*) between study variables in individuals with bipolar disorder (*n* = 53)

	EmoBio	
*Symptoms*		
YMRS	−0.08	ns
IDS-C^a^	0.07	ns
GAF-s	0.00	ns
*Functioning*		
GAF-f	0.12	ns
SFS total^b^	0.15	ns
UPSA-B^c^	0.48	*p* < 0.001
*Cognition*		
WASI	0.21	ns
MCCB total^c^	0.33	*p* = 0.017

^a^ *n* = 48, ^b^ *n* = 51, ^c^ *n* = 52

All bivariate associations between dosage of psychopharmacological treatment and emotion perception from body movements were non-significant (antipsychotics *r*_(*n* = 28)_ = 0.19, *p* = 0.335; antiepileptics *r*_(*n* = 12)_ = −0.50, *p* = 0.099; lithium *r*_(*n* = 16)_ = −0.28, *p* = 0.303; antidepressants *r*_(*n* = 11)_ = 0.46, *p* = 0.159).

## Discussion

In this study, we found that persons with BD have a small, but significant impairment in the ability to perceive emotions from body movement assessed with PLDs. This impairment was of a global nature, affecting all emotions to the same degree, and did not depend on participant sex. Further, the impairment was of a similar magnitude for individuals with BD I compared to those with BD II and was not impacted by having experienced psychotic symptoms.

The degree of impairment for this type of body language reading is, although present, not as severe as the one seen for schizophrenia. In our previous work (Vaskinn et al. 2016), persons with schizophrenia presented with a larger impairment compared to HC (Cohen’s *d* = 0.73) compared to an effect size of 0.40 in the current BD sample. This aligns with what we know about the neurocognitive and social cognitive performance in BD: it is intermediate between HC and individuals with schizophrenia (Bortolato et al. 2015; Bora and Pantelis 2016).

The magnitude of the impairment also aligns with the literature on social cognitive performance in different domains in BD. The performance level is the same as found for emotion perception using other types of stimuli during the euthymic phase in BD (*d* < 0.5: Samamé et al. 2012), but smaller than the deficit found for ToM tasks (*d* = 0.5–0.8: Samamé et al. 2012). The stimuli used in this study are simple, devoid of context and any information besides the emotional information of moving dots. It differs from stimuli of ToM tests where complex interpretation of the mental state (intentions, wishes, desires) of others is required. Task demand is therefore less, and the better performance compared to performance on ToM tasks is expected. It should also be noted that since there is heterogeneity within our BD sample, as seen in the standard deviations for the emotion perception task (see Table 2; Fig. 1), some individuals with BD have intact emotion perception.

Our sample as a whole is probably best characterized as mildly symptomatic, since less than half of the sample (43%) were euthymic according to standard criteria (Tohen et al. 2009). Body language reading performance was, however, not associated with global, depressive, or manic symptoms. This finding supports the notion that social cognitive impairment is a trait characteristic of the illness. This does not preclude that performance can be negatively affected by more severe levels of ongoing symptomatology. Studies of body language reading from PLDs in major depressive disorder (MDD) have given results in line with the idea of the “negative bias” of depression. Loi et al. (2013) found that currently depressed individuals had lower recognition rates of happy stimuli compared to individuals in remission from depression and HC, whereas Kaletsch et al. (2014) showed that individuals with MDD rated PLD scenes of human interactions more negatively than did HC. We cannot rule out that similar results will appear in studies of BD samples assessed during a mood episode. An investigation of this issue requires a different study design and is outside the scope of this article.

Another issue concerning clinical state is the association between psychopharmacological treatment and emotion perception performance. Although none of the associations were statistically significant, the sizes of the correlation coefficients suggest a strong relationship for antiepileptic and antidepressant medication (*r* = −0.50 and 0.46, respectively). The lack of statistical significance is probably due to the small sample. We do not find it likely, however, that our overall finding of impaired emotion perception in BD is due to the use of medication. These associations appear in the small subsample who received this type of psychopharmacological treatment, corresponding to approximately 20% of the BD sample (12 and 11 participants for antiepileptic and antidepressant medication, respectively). Although it is possible that social cognition performance is affected by psychopharmacological treatment, it is not likely that it causes the observed impairments in BD reported in the literature (Samamé et al. 2012; Bora et al. 2016). A proper examination of this research question requires a larger sample.

As expected, the performance on this social cognitive test was, using Cohen’s rules of thumb for interpretation of the strength of correlation coefficients, moderately and statistically significantly associated with neurocognition (performance on the MCCB battery). Others have also found that neurocognition and social cognition are related in BD (van Reenen et al. 2014). Although social cognition and neurocognition are considered to be two different constructs, they are related (Green et al. 2015). They encompass similar cognitive processes, but differ in the type of stimuli (social or non-social). Statistical modeling in different datasets has found that a separation between neurocognition and social cognition fit the data best (Allen et al. 2007; Bell et al. 2009). Further, neurocognition and social cognition are dependent on partly separable neural substrates (van Overwalle 2009). Therefore, neither of these cognitive domains can be reduced to the other.

Among the functional measures, emotional body language reading had small and non-significant associations with global functioning and self-reported social functioning. However, the association with functioning as assessed with a role-play task, functional capacity, was large and statistically significant. This is the same task that was predicted by an empirically derived social/emotional processing factor in a previous study of psychotic BD (Thaler et al. 2014). Our results both align and differ from the results of that study. Both studies are consistent with the hypothesis that social cognition predicts functioning in BD. Thaler et al. (2014) social cognition factor included complex stimuli, whereas the current study extends those findings by showing that even basic social cognition is associated with functioning. Further, whereas Thaler et al. (2014) only found this relationship in psychotic BD, our findings imply that this could be the case in the BD sample as a whole. Our results corroborate the findings of Lahera et al. (2012) that social cognition differentiates between low- and high-functioning individuals with BD. Our results suggest that even small emotion perception impairments can have negative functional consequences for individuals with BD. Taken together, these studies provide accumulating evidence that impaired social cognition is associated with worse functioning in BD.

Our findings are in line with an understanding of emotion perception impairment as a trait characteristic of BD. Emotions are important social signals, and their misinterpretation can have negative consequences for interpersonal functioning. As this can be expected to increase subjective feelings of stress and social rejection, emotion perception impairment could impact on the course of the illness by conferring risk for developing new affective episodes. Similarly, emotion perception impairment could be an endophenotype of BD, preceding the onset of illness (McKinnon et al. 2013). Studies that have found reduced emotion perception in first-degree healthy relatives of persons with BD (see for instance Seidel et al. 2012) support such a view. One could therefore speculate that emotion perception impairment is a vulnerability factor for developing BD in unaffected individuals, and for developing new affective episodes in individuals with BD.

Limitations of the study include the lack of a direct assessment of real-life functioning. Although our functional capacity measure, the UPSA-B, has ecological validity in the sense that it assesses real-world behavior, it is still administered in the test laboratory. Also, in statistical modeling of the path to functional outcome in schizophrenia, this measure behaves more like a measure of ability, with close connections to social cognition, than as a measure of functional outcome (Green et al. 2012). This could also be the case for BD. A truly ecological measure of functional outcome would require a different approach, such as video-ethnography where the person is video-taped in real life (Bromley et al. 2012). Another limitation is the current mood state of our sample. Our research design requires participants to be able to undergo our assessments, i.e., they are clinically stable. Testability does not, however, necessarily correspond to being in a euthymic state. Therefore, our sample is only partly euthymic. Thus, our research design does not enable a thorough investigation of the association between mood state and emotion perception. A further limitation is the sample size. Other results may appear with larger samples, for example for BD subgroupings.

In summary, we found a global impairment in the ability of individuals with BD to perceive emotions from bodily movement. The impairment was strongly associated with functional capacity. Our study implies that the perception of others’ emotions should be made part of clinical interventions and confirms a role for social cognition training in the treatment of BD (Lahera et al. 2013).

## References

[CR1] Allen DN, Strauss GP, Donohue B, van Kammen DP (2007). Factor analytic support for social cognition as a separable cognitive domain in schizophrenia. Schizophr Res.

[CR2] Baune BT, Malhi GS (2015). A review on the impact of cognitive dysfunction on social, occupational, and general functional outcomes in bipolar disorder. Bipol Disord.

[CR3] Bell M, Tsang HWH, Greig TC, Bryson GJ (2009). Neurocognition, social cognition, perceived social discomfort, and vocational outcomes in schizophrenia. Schizophr Bull.

[CR4] Birchwood M, Smith J, Cochrane R, Wetton S, Copestake S (1990). The Social Functioning Scale. The development and validation of a new scale of social adjustment for use in family intervention programs with schizophrenic patients. Br J Psychiatry.

[CR5] Bora E, Yucel M, Pantelis C (2009). Cognitive endophenotypes of bipolar disorder: a meta-analysis of neuropsychological deficits in euthymic patients and their first-degree relatives. J Affect Disord..

[CR6] Bora E, Pantelis C (2016). Social cognition in schizophrenia in comparison to bipolar disorder: a meta-analysis. Schizophr Res.

[CR7] Bora E, Bartholomeusz C, Pantelis C (2016). Meta-analysis of Theory of Mind (ToM) impairment in bipolar disorder. Psychol Med.

[CR8] Bortolato B, Miskowiak KW, Köhler CA, Vieta E, Carvalho AF (2015). Cognitive dysfunction in bipolar disorder and schizophrenia: a systematic review of meta-analyses. Neuropsychiatr Dis Treat..

[CR100] Bozikas VP, Kosmidis MH, Tonia T, Andreou C, Focas K, Karavatos A. Impaired perception of affective prosody in remitted patients with bipolar disorder. J Neuropsychiatry Clin Neurosci. 2007;19:436–40. 10.1176/jnp.2007.19.4.436 18070847

[CR9] Bromley E, Mikesell L, Mates A, Smith M, Brekke JS (2012). A video ethnography approach to assessing the ecological validity of neurocognitive and functional measures in severe mental illness: results from a feasibility study. Schizophr Bull.

[CR10] Cardenas SA, Kassem L, Brotman MA, Leibenluft E, McMahon FJ (2016). Neurocognitive functioning in euthymic patients with bipolar disorder and unaffected relatives: a review of the literature. Neurosci Biobehav Rev.

[CR11] Couture SM, Penn DL, Losh M, Adolphs A, Hurley R, Piven J (2010). Comparison of social cognitive functioning in schizophrenia and high functioning autism: more convergence than divergence. Psychol Med.

[CR12] Cullen B, Ward J, Graham NA, Deary IJ, Pell JI, Smith DJ (2016). Prevalence and correlates of cognitive impairment in euthymic adults with bipolar disorder: a systematic review. J Aff Disord..

[CR13] Demmo C, Lagerberg TV, Aminoff SR, Hellvin T, Kvitland LR, Simonsen C (2016). History of psychosis and previous episodes as potential explanatory factors for neurocognitive impairment in first-treatment bipolar I disorder. Bipol Disord..

[CR14] Dennis M, Francis DJ, Cirino PT, Schachar R, Barnes MA, Fletcher JM (2009). Why IQ is not a covariate in cognitive studies of neurodevelopmental disorders. J Int Neuropsychol Soc.

[CR15] Derntl B, Seidel E-M, Kryspin-Exner I, Hasmann A, Dobmeier M (2009). Facial emotion recognition in patients with bipolar I and bipolar II disorder. Br J Clin Psychol.

[CR200] First M, Spitzer R, Gobbon M, Williams J. Structured clinical interview for DSM-IV Axis I disorders, patient edition (SCID-P), version 2. New York: New York State Psychiatric Institute; 1995.

[CR16] Green MF, Penn DL, Bentall R, Carpenter WT, Gaebel W, Gur RC (2008). Social cognition in schizophrenia: an NIMH workshop on definitions, assessment, and research opportunities. Schizophr Bull.

[CR17] Green MF, Hellemann G, Horan WP, Lee J, Wynn JK (2012). From perception to functional outcome: modeling the role of ability and motivation. Arch Gen Psychiatry.

[CR18] Green MF, Horan WP, Lee J (2015). Social cognition in schizophrenia. Nat Rev Neurosci.

[CR19] Gunns RE, Johnston L, Hudson SM (2001). Victim selection and kinematics: a point light investigation of vulnerability to attack. J Nonverbal Behav.

[CR20] Heberlein AS, Adolps R, Tranel D, Damasio H (2004). Cortical regions for judgments of emotions and personality traits from point-light walkers. J Cogn Neurosci.

[CR21] Hellvin T, Sundet K, Vaskinn A, Simonsen C, Ueland T, Andreassen OA (2010). Validation of the Norwegian version of the Social Functioning Scale (SFS) for schizophrenia and bipolar disorder. Scan J Psychol..

[CR22] Hoertnagel CM, Biedermann F, Yalcin-Siedentopf N, Muehlbacher M, Rauch A-S, Baumgartner S (2015). Prosodic and semantic affect perception in remitted patients with bipolar I disorder. J Clin Psychiatry.

[CR23] Johansson G (1973). Visual perception of biological motion and a model for its analysis. Percept Psychophys.

[CR24] Kaletsch M, Pilgramm S, Bischoff M, Kindermann S, Sauerbier I, Stark R (2014). Major depressive disorder alters perception of emotional body movements. Front Psychiatry..

[CR25] Kern RS, Penn DL, Lee J, Horan WP, Reise SP, Ochsner KN (2013). Adapting social neuroscience measures for schizophrenia clinical trials, Part 2: trolling the depths of psychometric properties. Schizophr Bull.

[CR300] Kohler CG, Hoffman LJ, Eastman LB, Healey K, Moberg PJ. Facial emotion perception in depression and bipolar disorder: a quantitative review. Psychiatry Res. 2011;188:303–9. 10.1016/j.psychres.2011.04.019 21601927

[CR26] Kozlowski LT, Cutting JE (1977). Recognizing the sex of a walker from a dynamic point-light display. Percept Psychophys.

[CR27] Lahera G, Ruiz-Murugarren S, Iglesias P, Ruiz-Bennasar C, Herrería E, Montes JM, Fernández-Liria A (2012). Social cognition and global functioning in bipolar disorder. J Nerv Ment Dis..

[CR28] Lahera G, Benito A, Montes JM, Fernández-Liria A, Olbert CM, Penn DL (2013). Social cognition and interaction training (SCIT) for outpatients with bipolar disorder. J Affect Disord..

[CR29] Lembke A, Ketter T (2002). Impaired recognition of facial emotion in mania. Am J Psychiatry.

[CR30] Lewandowski KE, Cohen BM, Öngur D (2011). Evolution of neuropsychological dysfunction during the course of schizophrenia and bipolar disorder. Psychol Med.

[CR31] Loi F, Vaidya JG, Paradiso S (2013). Recognition of emotion from body language among patients with unipolar depression. Psychiatry Res.

[CR32] Martino DJ, Strejilevich SA, Fassi G, Marengo E, Igoa A (2011). Theory of mind and facial emotion recognition in euthymic bipolar I and bipolar II disorders. Psychiatry Res.

[CR33] Mausbach BT, Harvey PD, Goldman SR, Jeste DV, Patterson TL (2007). Development of a brief scale of everyday functioning in persons with serious mental illness. Schizophr Bull.

[CR34] McKinnon MC, Cusi AM, MacQueen GM (2013). Psychological factors that may confer risk for bipolar disorder. Cogn Neuropsychiatry.

[CR35] Mitchell RLC, Young AH (2014). Theory of mind in bipolar disorder, with comparison to the impairments observed in schizophrenia. Front Psychiatry.

[CR36] Mohn C, Sundet K, Rund BR (2012). The norwegian standardization of the MATRICS (measurement and treatment research to improve cognition in schizophrenia) consensus cognitive battery. J Clin Exp Neuropsychol.

[CR37] Nuechterlein KH, Green MF, Rund BR, Sundet K (2009). MATRICS consensus cognitive battery. Norwegian version.

[CR38] Okruszek L, Haman M, Kalinowski K, Talarowska M, Becchio C, Manera V (2015). Impaired recognition of communicative interactions from biological motion in schizophrenia. PLoS ONE.

[CR39] Pedersen G, Hagtvedt KA, Karterud S (2007). Generalizability studies of the global assessment of functioning-split version. Compr Psychiatry.

[CR40] Pinkham AE (2014). Social cognition in schizophrenia. J Clin Psychiatry.

[CR41] Rush AJ, Gullion CM, Basco MR, Jarrett RB, Trivedi MH (1996). The inventory of depressive symptomatology (IDS): psychometric properties. Psychol Med.

[CR42] Samamé C, Martino DJ, Strejilevich SA (2012). Social cognition in euthymic bipolar disorder: systematic review and meta-analytic approach. Acta Psychiatr Scand..

[CR43] Samamé C (2013). Social cognition throughout the three phases of bipolar disorder: a state-of-the-art overview. Psychiatry Res.

[CR44] Sanchez-Moreno J, Martinez-Aran A, Tabarés-Seisdedos R, Torrent C, Vieta E, Ayuso-Mateos JL (2009). Functioning and disability in bipolar disorder: an extensive review. Psychother Psychosom.

[CR45] Seidel EM, Habel U, Finkelmeyer A, Hasmann A, Dobmeier M, Derntl B (2012). Risk or resilience? Empathic abilities in patients with bipolar disorder and their first-degree relatives. J Psychiatr Res.

[CR46] Simon GE (2003). Social and economic burden of mood disorders. Biol Psychiatry.

[CR47] Simonsen C, Sundet K, Vaskinn A, Birkenaes AB, Engh JA, Faerden A (2011). Neurocognitive dysfunction in bipolar and schizophrenia spectrum disorders depends on history of psychosis rather than diagnostic group. Schizophr Bull.

[CR48] Spitzer RL, Williams JB, Kroenke K, Linzer M, III deGruy FV, Hahn SR (1994). Utility of a new procedure for diagnosing mental disorders in primary care. The PRIME-MD 1000 study. JAMA.

[CR49] Thaler NS, Sutton GP, Allen DN (2014). Social cognition and functional capacity in bipolar disorder and schizophrenia. Psychiatry Res.

[CR50] Tohen M, Frank E, Bowden CL, Colom F, Ghaemi SN, Yatham LN (2009). The International Society for Bipolar Disorders (ISBD) task force report on the nomenclature of course and outcome in bipolar disorders. Bipol Disord..

[CR51] van Overwalle F (2009). Social cognition and the brain: a meta-analysis. Hum Brain Map..

[CR52] van Reenen TE, Rossell SL (2013). Auditory-prosodic processing in bipolar disorder; from sensory to emotion. J Affect Disord..

[CR53] van Reenen TE, Meyer D, Rossell SL (2014). Pathways between neurocognition, social cognition and emotion regulation in bipolar disorder. Acta Psychiatr Scand..

[CR54] Vaskinn A, Sundet K, Friis S, Simonsen C, Birkenaes AB, Engh JA (2007). The effect of gender on emotion perception in schizophrenia and bipolar disorder. Acta Psychiatr Scand..

[CR55] Vaskinn A, Sundet K, Østefjells T, Nymo K, Melle I, Ueland T (2016). Reading emotions from body movement: a generalized impairment in schizophrenia. Front Psychol..

[CR56] Wechsler D (2007). Wechsler Adult Intelligence Scale, WAIS-III, Norwegian manual.

[CR57] World Health Organization. DDD. Definition and general considerations. www.whocc.no/ddd/definition_and_general_considera. Accessed 30 Nov 2016.

[CR58] Young RC, Biggs JT, Ziegler VE, Meyer DA (1978). A rating scale for mania: reliability, validity and sensitivity. Br J Psychiatry.

